# Determining the level of condom use and associated factors among married people in Tshwane District of South Africa

**DOI:** 10.11604/pamj.2021.40.11.26681

**Published:** 2021-09-03

**Authors:** Lindiwe Priscilla Cele, Setati Vuyani, Mmampedi Huma

**Affiliations:** 1 Department of Public Health, School of Health Care Sciences, Sefako Makgatho Health Sciences University, Pretoria, South Africa

**Keywords:** Condom use, married people, trust, HIV transmission rate, sero-discordance

## Abstract

**Introduction:**

despite the wide availability of free male condoms in South Africa, high rates of new HIV transmissions are reported to occur among married couples. The aim of this study was to determine the level of condom use among the married people and to assess the factors associated with condom use in the Tshwane district of the Gauteng province.

**Methods:**

a cross-sectional study was conducted among 325 clients accessing health care services at the Steve Biko Academic Hospital. A self- administered questionnaire was used to collect data, which were entered onto an Excel spreadsheet and imported into Epi info version 7 for analysis. A logistic regression model was used to investigate the association between condom use and the explanatory variables. The odds ratio was used to measure the strength of the associations. The 95% CI and a cut-off point of 0.05 for the p-value were used to indicate statistical significance.

**Results:**

the mean age of the participants was 41.6 years (SD=7.7). Two hundred and seventy-six (276; 85%) of the 325 participants reported not using condoms. Trust, doing regular HIV testing, and refusal by the husband were among the reasons given for not using condoms. In this study it was found that, the level of education, age and employment status were the determinants of condom use.

**Conclusion:**

the level of condom use was low and sero-discordance was found to be the primary motivator for condom use. This study recommends the strengthening of and the widespread implementation of the CVCT service.

## Introduction

**Background/rationale:** condom use remains a critical component in the prevention of the Human Immunodeficiency virus (HIV) and other sexually transmitted infections (STIs). It is also prevents many unplanned pregnancies. To be effective, condoms have to be used consistently and correctly [1]. Following a political declaration by a number of countries in 2016, it was projected that an estimated 20 billion additional condoms would be made available globally, an initiative that would see an estimated 3.9 million new HIV infections averted globally by 2020 [2]. As a contraceptive, condoms are said to have relatively fewer-side effects and to be easier to use than other contraceptive methods [3].

In sub-Saharan Africa (SSA), the level of condom use is said to be low, yet the region is reported to have high rates of transmission of HIV and STIs. The influence of age, gender, poverty, and a lack of or insufficient information on condom use have been reported by some studies, especially in the rural settings of this region, to be responsible for the low level of condom use [4]. In South Africa, male condoms are freely and widely available as part of the national public sector condom programme. However, inconsistent and incorrect use of condoms and the possible interruption of use in long-term relationships are said to be limiting the success of the programme. According to the South African National Survey of 2012, consistent condom use was lowest among people aged 50 years and above [5].

Marriage can be thought of as a safe haven when it comes to the transmission of STIs and HIV. However, the number of new HIV infections is said to be higher among married and cohabiting couples, with 80% of new infections in SSA said to occur among married women [5, 6]. The increased frequency of sex and low condom use among stable relationships such as a marriage are said to be the cause of the risk of HIV transmission among married people [7]. Married individuals in SSA are especially rendered more susceptible to HIV infection due to the general prevalence of HIV and the gender inequalities that exist in most countries of this region [8]. Malawi is one such country where this gender inequality characterizes most marriages, a fact which has led Malawian women to perceive marriage as a risk factor for HIV infection [7]. In a study that was done in Nigeria, marriage was given as a reason for not using condoms [9].

The widespread association of condom use with promiscuity and unfaithfulness is said to be a major contributor to the low levels of condom use among the married couples [4]. Others attribute the low prevalence of condom use to neglect of this group in the promotion of consistent condom use [10]. In some studies that were conducted in South Africa, marriage was found to be a barrier to condom use, which may relate to the desire of the couples to have children, making negotiating condom use difficult [11, 12]. Studies reporting a low prevalence of condom use among married couples include one that was conducted among married and cohabiting women in South Africa. In this study, the prevalence of condom use was found to be 16.7%, and this was after a 6-month reference period. According to this study, the main reason for this low level of use was the trust that these women claim exists between them and their partners [10].

Culture and male dominion have been cited as barriers to condom use in marriage, especially in SSA, where condom use remains predominantly under control of men [4]. The cultural norms that prevail in marriage and sexual relationships give menthe authority in decision-making, and this may afford them the leeway to resist using condoms, which causes frustrations among the women [10, 11]. In one Ugandan study, family planning was used to justify condom use due to a lack of the non-barrier contraceptives. This is said to have assisted in evading accusations of using condoms to prevent STIs [13]. However, with the eventuality of sero-discordance among married couples, condom use has become increasingly necessary within marriage. South Africa is said to have one of the highest HIV prevalence rates in the world, with prevalence among the individuals in the 50-54 year age group reported to be rising and said to have been 13% in 2012 [2, 5]. This is presumably the age range of married people. Moreover, the country is also said to have one of the highest rates of violence against women in the world, with reported drastic increases in the murder rate for women and sexual offences against women jumping by 117% and 53% respectively between 2015 and 2016 [14]. Hence, this study aimed at determining the prevalence of condom use and to document contributing factors, including behaviour and the attitude towards condom use among married people in Tshwane district.

**Objectives:** the study objectives were to determine the level of condom use and describe the factors contributing to condom use among married people in the Tshwane district.

## Methods

**Study design:** this was a descriptive cross-sectional study utilizing the method of primary data collection.

**Study setting:** the study was conducted at Steve Biko Academic Hospital (SBAH) in Pretoria, Tshwane district of the Gauteng province. Pretoria is about 54 km from OR Tambo, with two academic hospitals, one tertiary and two district hospitals. The SBAH is an academic, tertiary hospital, which renders specialized and highly specialized services to medically referred patients from health facilities in the Gauteng and other provinces such as Limpopo and Mpumalanga. The hospital has 832 beds and has 22 out-patient clinics that specialize in oncology, specialized surgery, general surgery, high risk pregnancy, fertility treatment, cardiology, neurology, psychiatry, Ear-Nose and Throat (ENT) and other conditions. These clinics attend to more than 2000 patients per month [15]. The study was conducted in out- patient clinics and out-patient pharmacy. The researcher sampled 4 out-patient clinics and out-patient pharmacy.

**Participants:** the study participants recruited among clients that were seeking health care from the clinics and the outpatient departments of the SBAH between the months of June and July 2018. Prospective participants were eligible if they were married and showed no sign of being critically ill. Those who willing to participate were asked to sign a consent form before proceeding with the interviews. Those that were referred from provinces outside Tshwane district were excluded from participation.

**Variables:** the study collected the socio- demographic data such as age, gender, race, number of years married, residential setting (urban versus rural), employment status, level of education and religion. Other variables included questions that were designed to determine the participants´ attitude and behaviour patterns relative to condom use, circumstances that would cause the participants to use condoms and the reasons for not using condoms. These include questions that focused on among others, the ability to negotiate and initiate condom use, and their feelings when a partner asked to use a condom.

**Data sources:** the study utilized the primary method of data analysis through conducting interviews.

### Bias

**Selection bias:** this study used a convenient sample, rendering the sample unrepresentative of the source population. To approach representativeness, the study determined to enrol more participants than the calculated sample size, however, this number could not be reached during the study period.

**Information bias:** condom use was self-reported, and therefore prone to information inaccuracies or biases due to participants giving answers that would be considered socially acceptable. To reduce this bias, the study ensured confidentiality by making the questionnaires to be anonymized.

**Study size:** for sample size determination, the study used the Raosoft sample size calculator, which is available online. Based on the total annual client population of 24,000 that are seen at the clinics and the outpatients´ departments, and assuming the 95% confidence interval, the sample size was determined to be 379. To compensate for possible low response rate and missing values, a 10% buffer of participants were included to make a final sample size of 417 participants. A total of 325 participants had been enrolled after six weeks of recruitment.

**Quantitative variables:** the mean and the standard deviations were used to present the continuous variables, age and the years of marriage were presented as the mean and the standard deviation, and these were displayed using frequency tables. These variables were converted into categories and stratified by condom use, and presented as percentages and proportions along with the other categorical data such as sex, level of education, employment status and religion.

**Statistical methods:** the study conducted univariate analysis on all the variables. Binary logistic regression analysis was conducted to identify determinants of condom use. Some socio-demographic variables were dichotomized and used as the explanatory variables. The odds ratio (OR) >1 indicated a positive association, and a p value of <0.05 signified statistical significance of the association. The data were displayed on frequency tables.

## Results

**Descriptive data:** a total of 325 participants were enrolled in this study, and their ages ranged from 30 to 60 years, with a mean of 41.6 years (SD=7.7). The majority (83%) were in the 30-49 year age group, and most (65%) were black, (84.9%) were female, 91.4% were Christian, and 84.5% were living in an urban residence. The mean duration of the marriages was 13.2 years (SD=9.3) with the majority (41.2 %) reporting to have been married for less than 10 years. More than half (64%) had attained a tertiary level of education, while only 1% reported having had no schooling. About half (50.5%) of the participants reported being fully employed, with 4% and 13% reporting part-time and self-employment, respectively (Table 1).

**Table 1:** frequency distribution of participants´ socio-demographic characteristics stratified by condom use (n=325)

	Condom use		
Characteristic	Yes (n=49)	No (n=276)	Total (%)
**Gender**			
Female	38 (77.6)	198 (71.7)	276 (84.9)
Male	11 (22.4)	78 (28.3)	89 (15.1)
**Age in years**			
Mean (SD)	41.6 (7.7)		
**Age group**			
30-39	21 (43.0)	121 (43.8)	142 (43.7)
40-49	21 (43.0)	106 (38.4)	127 (39.1)
50-59	7 (14.0)	43 (15.6)	50 (15.4)
60+	0 (0.0)	6 (2.2)	6 (1.8)
**Employment**			
Employed	20 (40.8)	144 (52.1)	164 (50.5)
Part time	3 (6.1)	11 (4.0)	14 (4.3)
Self-employed	9 (18.4)	32 (11.6)	41 (12.6)
Unemployed	17 (36.7)	89 (32.2)	106 (32.6)
**Level of education**			
No schooling	1 (2.0)	3 (1.1)	4 (1.0)
Primary	2 (4.0)	2 (1.0)	4 (1.0)
Secondary	21 (43.0)	89 (32.0)	110 (34.0)
Tertiary	25 (51.0)	182 (65.9)	207 (64.0)
**Residence**			
Urban	40 (81.6)	241 (87.3)	281 (84.5)
Rural	9 (18.4)	35 (12.7)	44 (13.5)
**Race**			
Asian	1 (2.0)	7 (2.5)	8 (2.0)
Black	36 (74.0)	175 (63.4)	211 (65.0)
Coloured	2 (4.0)	24 (8.7)	26 (8.0)
White	10 (20.0)	70 (25.4)	80 (25.0)
**Religion**			
Christian	43 (88.0)	254 (92.0)	297 (91.4)
Muslim	4 (8.0)	8 (2.9)	12 (3.7)
Other	2 (4.0)	14 (5.1)	16 (4.9)
**Years married**			
Mean (SD)	13.2 (9.3)		
**Group years married**			
<10	18 (37.0)	116 (42.0)	134 (41.2)
10-20	20 (41.0)	111 (40.2)	131 (40.3)
>20	11 ((22.0)	49 (17.8)	60 (18.5)

### Outcome data

**Condom use:** Table 1 presents the level of condom use among the participants. The results indicate that 15% (49) of the 325 participants affirmed using condoms. Condom use was high among: females (78%), Blacks (74%), individuals between the ages of 30 and 49 years (43%), employed individuals (41%), people with a tertiary education (51%), people living in an urban residence (82%), Christians (88%), and people married for between 10 and 20 years (41%). Of the 72% non-condom users, the majority (85%) were also female, (72%) black (44%), in the 30-39-year age group (52%), employed (66%), with a tertiary education (87%) living in an urban residence (42%) married for less than 10 years.

**Reasons for non-condom use:** a total of 276 participants provided a reason for not using a condom, and of these, sixty-three (63) were excluded from the analysis because they had indicated that they used condoms. Of the remaining 213, 29% did not state a reason for not using a condom. The reasons included (32%) that they trusted their partners, 9% who wanted to conceive (9%). Of the female participants, 7.5% reported refusal by their husbands to use a condom, 6% said that they did regular HIV testing, and 3% gave religion as the reason. About 10% stated that there was no reason to use a condom (Table 2).

**Table 2:** reasons for not using a condom stratified by gender (n=276)

Reason	Frequency (%)		
	Total (n=213)	Female (n=152)	Male (n=61)
Trust each other	69 (32.0)	45 (30.0)	24 (39.3)
Want to conceive	18 (9.0)	13 (9.0)	5 (8.2)
We do regular HIV testing	12 (6.0)	9 (6.0)	3 (6.4)
There is no need to use condoms	22 (10.0)	11 (7.2)	11 (18.0)
Husband refuses to use a condom	16 (7.5)	16 (10.5)	0 (0.0)
Religious purposes	7 (3.0)	5 (3.3)	2 (3.0)
Allergic to latex	3 (1.0)	3 (1.9)	0 (0.0)
Better without condom	1(0.5)	0(0.0)	1 (1.6)
Both HIV negative	1 (0.5)	1 (0.7)	0 (0.0)
Cannot use condom with my wife	1 (0.5)	1 (0.7)	0 (0.0)
We do not like using it	1 (0.5)	1 (0.7)	0 (0.0)
The other partner is not ready to use it	1 (0.5)	1 (0.7)	0 (0.0)
No reason given	61 (29.0)	46 (30.3)	15 (24.5)

* 63 responses excluded from the 276 participants

**Circumstances that might cause condom use among the participants:** Table 3 shows the circumstances that the participants indicated would cause them to use condoms, and where it can be noted that of the 198 participants who responded, the majority (39.1%) stated that they would use condoms if there was cheating or a suspicion of cheating by the partner, (13%) stated illness, (5.1%) menstruation and 2.5% stated that they would use condoms on a doctor´s recommendation. A few (2%) stated that they would use a condom if the partner tested positive for the HIV, and 1.0% stated that a partner´s refusal to test for HIV was a circumstance that might cause them to use a condom.

**Table 3:** circumstances that can make participants use a condom (n=276)

Circumstance	Frequency	%
When there is cheating/suspicion of cheating	108	40.0
Preventing a pregnancy	20	7.4
Illness	36	13.3
If a spouse tests positive for HIV	5	1.9
If the partner refuses to test for HIV	3	1.1
When the partner is menstruating	14	5.2
On a doctor´s recommendation	7	2.6
No circumstance given	78	28.5
**Total**	**270**	**100**

**Behaviour and attitude towards condom use:** Table 4 presents the participants´ responses to the behaviour and attitude questions. The majority (68.5%) found using condoms within a marriage acceptable, with 92% affirming having access to condoms. About 73.2% indicated that they could ask a partner to use a condom, with 6%, most of whom were male participants, indicating that they would be angry if the partner asked to use a condom. The majority (41%) stated that they were not worried about HIV transmission, but 69.6% of the participants indicated that they could consider using condoms in future.

**Table 4:** attitudes and behavior towards condom use among participants not using condoms, (n=276)

	Total	Female	Male
	Frequency (%)		
**Do you think it is acceptable to use a condom within the marriage?**			
Yes	189 (68.5)	141 (71.2)	48 (61.5)
No	87 (31.5)	57 (28.8)	30 (38.5)
**Do you have access to condoms?**			
Yes	256 (92.2)	185 (93.4)	71 (91.0)
No	20 (7.2)	13 (6.6)	7 (9.0))
**Can you ask your partner to use a condom?**			
Likely	202 (73.2)	147 (74.2)	55 (70.5)
Unlikely	74 (26.80	51 (25.8)	23 (29.5)
**How would you feel if your partner asked to use a condom?**			
Would not mind	180 (65.0)	135 (68.2	45 (57.7)
Would be angry	16 (6.0)	5 (2.5)	11 (14.1)
Not sure	80 (29.0)	58 (29.3)	22 (28.2)
**How worried are you about getting HIV?**			
Very Worried	97 (35.1)	70 (35.3)	27 (34.6)
Worried	67 (24.3)	46 (23.2)	21 (26.9)
Not worried	112 (40.6)	82 (41.4)	30 (38.5)
**Would you consider using a condom in the future?**			
Yes	192 (69.6)	142 (71.7)	50 (64.1)
No	19 (6.9)	13 (6.6)	6 (7.7)
Maybe	65 (23.5)	43 (21.7)	22 (28.2)
**Total**	**276**	**198**	**78**

**Factors associated with condom use in marriage:** Table 5 displays the results from a logistic regression analysis. From this table it can be noted that condom use was associated with level of education (OR 3.53, 95% CI 0.81-15.29), age (OR 1.29, 95% CI0.55-3.05) and employment status (OR 1.11, 95% CI0.58-2.12). These associations were not statistically significant, with p values 0.07; 0.55 and 0.74 respectively.

**Table 5:** demographic determinants of condom use (N=325)

Characteristic	Condom Use			
	Yes	No	OR (95% CI)	p-value
**Age**	****	****	****	****
≤49	42	227	1.29 (0.55-3.05)	0.554
>49	7	49		
**Gender**				
Female	38	198	0.73 (0.36-1.57)	0.400
Male	11	78		
**Employment status**				
Employed	32	187	1.11 (0.58-2.12)	0.736
Unemployed	17	89		
**Level of education**				
Low	3	5	3.53 (0.81-15.29	0.073
High	46	271		
**Race**				
Black	35	175	0.62 (0.32-1.23)	0.173
Other	13	101		
**Years of marriage**				
≤20	38	227	0.75 (0.36-1.56)	0.435
>20	11	49		
**Total**	**49**	**276**	****	****

## Discussion

**Key results:** the level of condom use among the married people found in this study is low (15%). This is despite the majority of the participants in the current study affirming access to condoms, with more than half (69%) indicating that they found condom use in marriage acceptable, others indicating that they could ask their partners to use condoms, and still others stating that they would not mind if the partner asked to use a condom. Similar results have been found in other studies that were conducted in Kenya, Nigeria, and South Africa [10, 16, 17]. Interestingly, more than half of the participants indicated that they would consider using condoms in future.

Condom use was higher among those younger than 50 years and that had been married for less than 20 years. This is consistent with the findings of other studies conducted in Eastern Zimbabwe and Kwa-Zulu Natal, in which the level of condom use decreased with increasing age [5, 10, 18, 19]. The study also determined the reasons for not using the condom among the 85% that had indicated to be not using condoms. The most common reason was trusting the partners, which was also a common finding in other studies [10, 20, 21]. Other reasons were the husband´s refusal to use condoms (7.5%), doing regular testing to check the HIV status (6%), and 10% who thought there was no need for condom use in marriage (10%).

This study further examined the attitudes and behaviour of the participants towards condom use and the circumstance that could make participants to use a condom. The results indicated that 41% of the participants were not worried about HIV whilst others, mostly males, indicated that they would be angry if the partner were to ask for a condom partner. Logistic regression revealed that younger age (≤ 49 years old), higher education, and being employed were predictors of condom use in the current study, findings that have been observed in other studies [3, 16, 22].

**Limitations:** this study used a convenient sample, which rendered it unrepresentative. The sample was small, which might have contributed to the wider confidence intervals and failure of the observed associations to achieve statistical significance.

**Interpretation:** doing regular testing to check the HIV status, according to this study, indicates that infection with HIV is the main driver for condom use among some married couples. In studies that were conducted in South Africa and Uganda, consistent condom use was higher among couples where both or one partner were thought to be HIV positive [23, 24]. Additionally, being not worried about HIV indicates low HIV-related risk perception and this could be the reason behind the low level of condom use among participants in this study, as other studies have found an association between a high HIV risk perception and consistency in condom use [23, 24]. Conversely, sero-discordance appears to have been the primary motivator for using condoms among married people in this study. These factors are an indication of the increased vulnerability of married people to HIV infections, as well as the shortcomings that traditional approaches to HIV prevention have in meeting the needs of married couples, especially those of women. Refusal by husbands to use a condom, and the anger should a partner ask to use a condom as expressed by mainly the male participants, is an indication of the male dominion that still exists among some communities. This according to some study causes frustration among women and could compromise the efforts of women to protect themselves from acquiring HIV infections [10, 25].

**Generalizability:** due to the unrepresentative nature of the sample, this study results cannot be generalized to the whole population.

## Conclusion

The prevalence of condom use among the married people in this study was low. Employment status, age and level of education were the determinants of condom use. The low HIV- related risk perception seems to have resulted in a failure to use condoms, and conversely, sero-discordance appears to have been the primary motivator for using condoms among married couples. These factors indicate the increased vulnerability of married people to HIV infections. The findings are an indication of the shortcomings that traditional approaches to HIV prevention have in meeting the needs of married couples, especially those of women. In South Africa, there are few voluntary counselling and testing (CVCT) services for couples. This is despite the findings from studies indicating a high prevalence of sero-discordance (29.5%) and low levels of knowledge, as indicated by 70% of the couples, who did not know about discordance. This study therefore recommends the strengthening of the CVCT and the wide implementation of this service, where feasible. Focus should be on the strengthening of the recruitment efforts targeting couples, counselling for both members (individually and together), and support for coping and risk management, especially when the seropositive partner is the woman. In a Ugandan study, official letters of invitation were issued to promote male attendance of CVCT, and testing in antenatal care. The results were a significant improvement in male participation, with more than half of the women returning with their partners.

### What is known about this topic

- There is a high level of sero-discordance and a poor understanding of sero-discordance among most married people;
- The CVCT uptake is low;
- The focus of the National Strategic Plan on the prevention of HIV/AIDS (NSP 2017-2022), including the previous NSPs, is not inclusive of married couples.

### What this study adds

- Some married people conduct regular HIV testing in place of using condoms;
- The level of knowledge on sero-discordance should be increased;
- CVCT should be strengthened and implemented widely. Ways to engage partners, more especially male partners, should be sought.
